# Whole genomic analysis of a potential recombinant human adenovirus type 1 in Qinghai plateau, China

**DOI:** 10.1186/s12985-020-01387-x

**Published:** 2020-07-22

**Authors:** Juan Yu, Shengcang Zhao, Huaxiang Rao

**Affiliations:** 1 Department of Basic Medical Sciences, Changzhi Medical College, 161 Jiefang East Street, Changzhi, 046000 China; 2 Center of Hygiene Inspection, Qinghai Center for Disease Control and Prevention, 55 Bayi Middle Road, Xining, 810007 China; 3 Department of Public Health and Preventive Medicine, Changzhi Medical College, 161 Jiefang East Street, Changzhi, 046000 China

**Keywords:** Human adenovirus (HAdVs), Whole genome sequencing, Recombination analysis

## Abstract

Human adenoviruses (HAdVs) are prevalent in patients with respiratory infections, in which recombination has important implications for viral detection and pathogenicity. However, less HAdVs recombination was reported in Qinghai plateau. In this study, we obtained an HAdV-C strain (QH-1665/2018) isolated from an infant aged one month with influenza-like illness in Qinghai Province in 2018. The whole genome sequence was generated by next-generation sequencing, and compared with that of other HAdV-C strains available in public. The strain QH-1665/2018 genome is comprised of 36,014 nucleotides and encoded 36 putative proteins. Phylogenetic analysis of complete HAdV genomes and 3 major antigen genes (penton, hexon and fiber) showed that strain QH-1665/2018 was clustered into HAdV-1 [P1H1F1]. Recombination analysis based on the RDP4 package and SimPlot software showed that QH-1665/2018 was a recombinant involving HAdV-1, HAdV-2 and HAdV-5, which was then re-confirmed by phylogenetic analysis. Our results suggest that HAdV-C recombination is highly complex, should be focused on, and the epidemiological and virological surveillance should be strengthened in Qinghai Province.

## Introduction

Adenovirus is a non-enveloped, double-stranded DNA virus belonging to the Adenoviridae family, that can cause infections related to the respiratory tract, gastrointestinal tract and eyes in humans [1]. Although HAdV infection is mild and self-limited in the healthy individuals, life-threatening disease can occur in immune-compromised patients [2, 3]. Currently, human adenoviruses (HAdVs) are classified into seven groups (HAdV-A to HAdV-G), including 52 serotypes and 90 genotypes [4]. Among which, HAdV-C contains HAdV-1, HAdV-2, HAdV-5, HAdV-6, HAdV-57 and HAdV-89, which are common etiological factors in respiratory infections, especially for severe bronchiolitis or pneumonia in children [5, 6].

Complete genomic data have enhanced the understanding of HAdV epidemiology and is an important way to recognize the recombination in HAdV pathogens. In recent years, genetic recombinant of HAdV-C has also been sporadically identified in humans [4, 7, 8]. However, until now, little data on HAdV recombination have been reported in Qinghai Province. Here, we describe the characterization of an HAdV-1 recombinant (QH-1665/2018) isolated from an infant aged one month with influenza-like illness in Qinghai Province in 2018. This information would enhance the understanding of recombination of HAdV-C, and might assist with effective prevention and control of respiratory adenovirus infection in Qinghai Province.

## Materials and methods

### Sample collection and identification

The Qinghai adenovirus strain(QH-1665/2018) was isolated from an outpatient, whom was one month old and diagnosed with an influenza-like illness at Women’s and Children’s Hospital of Qinghai Province on November 26, 2018. Nasopharyngeal swab specimen of this patient was HAdV positive as detected using our previous methods [9].

### Virus isolation

The HAdV positive samples were inoculated onto human laryngeal epidermoid cancer cells (HEp-2) cultured with DMEM containing 2% FBS for virus isolation. After incubation for 7 days, if no cytopathic effect (CPE) appeared, the cultures were collected and two additional passages were conducted; if CPE appeared, the cultures were passaged again to confirm the presence of virus. QH-1665/2018 caused adenovirus-like CPE of HEp-2 cells, and the cultures underwent three passages to obtain high-titer stocks. The virus-infected cells and supernatant were then collected and used for subsequent genome sequencing.

### Whole-genome sequencing

Whole-genome sequencing of HAdV strain QH-1665/2018 was performed on Illumina HiSeq Xten platform (PE 150) by BioGerm (Shanghai, China), and the complete genome of HAdV was then assembled by SPAdes software.

### Phylogenetic analysis

The HAdV nucleotide sequences were analyzed by using BioEdit version 7.0.4.1 and NCBI BLAST software (http://blast.ncbi.nlm.nih.gov/). MEGA 6.06 software was used for phylogenetic analysis of aligned sequences. The phylogenetic tree was generated using the Maximum Likelihood (ML) algorithm. The credibility of the phylogenetic tree was tested by applying a bootstrap test with 1000 replications [10].

### Recombination analysis

The Recombination Detection Program (RDP) package Beta 4.100 was used for identification of recombinant sequences in default mode. A recombination event with a significance of *p* < 0.01 in at least three out of seven selected algorithms: RDP, GENECONV, BootScan, Maxchi, Chimaera, SiScan, and 3Seq, was considered to be reliable. Recombination events were then confirmed and visualized with SimPlot Version 3.5.1. Bootscan analysis was used to test potential recombination events in the default settings. The sequence of the QH-1665/2018 strain was used as the query sequence and compared to those of other HAdV-C isolates with a sliding window of 200 bp.

## Results

### Complete genomic characterization

**Fig. 1:**
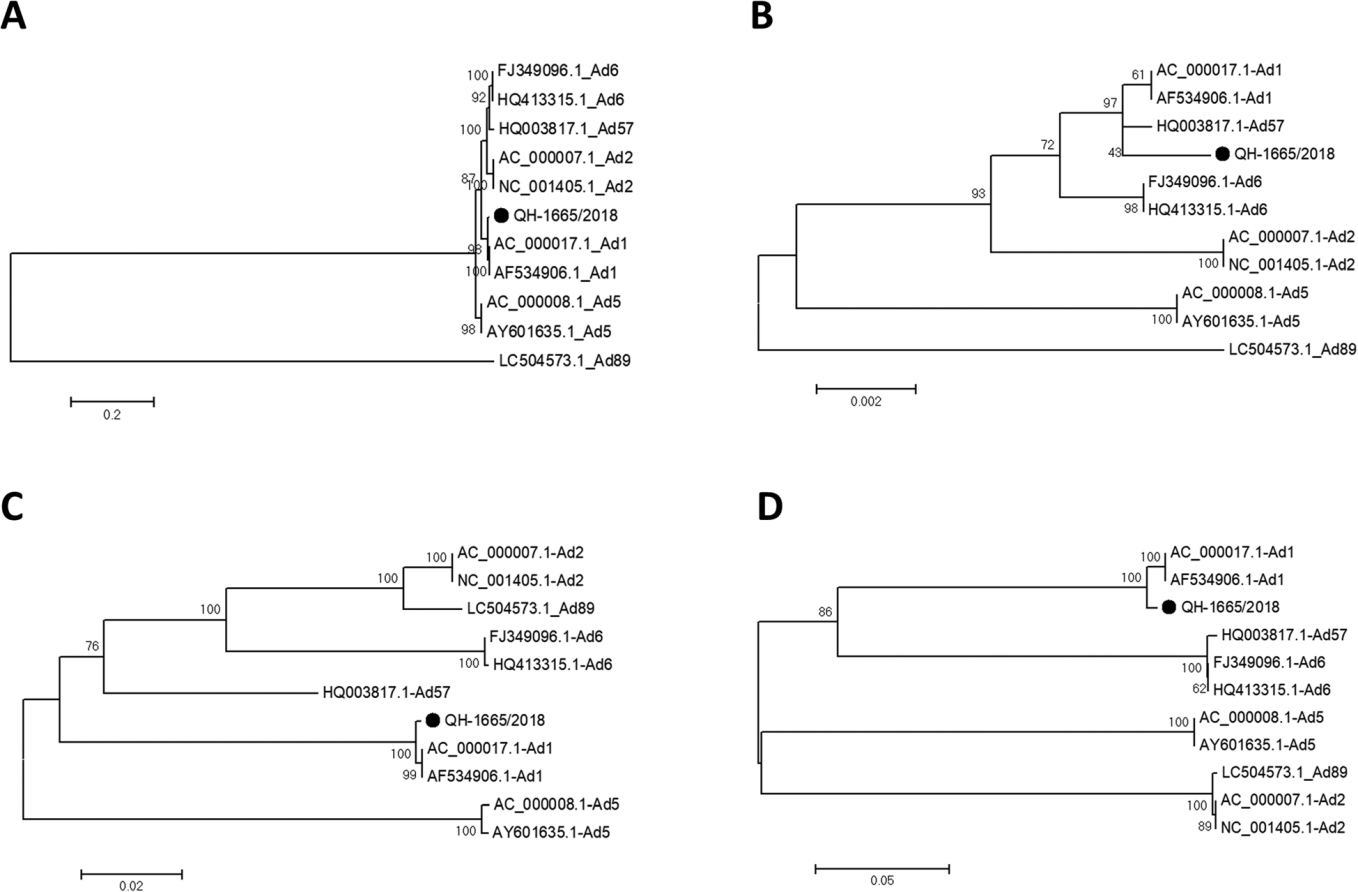
Phylogenetic analysis of HAdVs based on the complete genome (**a**), penton (**b**), hexon (**c**) and fiber gene (**d**). The trees were constructed using the Maximum Likelihood (ML) method of MEGA 6.06 with 1000 bootstrap trials. QH-1665/2018 highlighted with a black dot was characterized in this study

Using next-generation sequencing, the full-length genomic sequence of the Qinghai HAdV isolate (QH-1665/2018) was determined and deposited in GenBank (accession number MN737436). The genome is comprised of 36,014 nucleotides with a GC content of 55.34% and encodes 36 putative proteins. Whole genome phylogenetic analysis of complete HAdV genomes illustrated that strain QH-1665/2018 was clustered into HAdV-1, and phylogenetic analysis of penton, hexon and fiber genes showed that the 3 major antigen genes were classified into P1, H1, and F1 (Fig. 1).

### Homology analysis

Comparison with the complete genome sequences of the six HAdV-C strains (HAdV-1, HAdV-2, HAdV-5, HAdV-6, HAdV-57 and HAdV-89) showed that QH-1665/2018 was conserved, sharing the highest nucleotide identity (99.39%) with HAdV-1. According to Zhang’s method [4], we compared the nucleotide sequence identity of coding regions within QH-1665/2018 with that of HAdV-C representative strains. The nucleotide sequences of the penton, hexon and fiber genes showed the highest nucleotide identity with HAdV-1, which was consistent with that of the phylogenetic analysis. Furthermore, E1A, pIX, Iva2, DNA polymerase, pIIIa, V, pX, pVI, DBP, 100 K, pVIII, E3 and fiber showed the highest sequence similarity with HAdV-1. Interestingly, we found that the 52 K gene showed the highest sequence similarity with HAdV-2, the E4 gene showed the highest sequence similarity with HAdV-5, the pTP and protease gene showed the highest sequence similarity with HAdV-6, the E1B region showed the highest sequence similarity with HAdV-57, and the pVII gene showed the highest similarity with both HAdV-6 and HAdV-57. These results suggested that QH-1665/2018 might be a recombinant strain (Table 1).

**Table 1:** Nucleotide sequence identities between QH-1665/2018 and HAdV-C representative strains

Region	Nucleotide identity (%)					
	QH-1665/2018					
	HAdV-C1	HAdV-C2	HAdV-C5	HAdV-C6	HAdV-C57	HAdV-C89
E1A	**99.43**	98.74	99.08	98.74	98.62	98.74
E1B	99.06	99.24	97.69	98.18	**99.28**	98.24
pIX	**100.00**	99.53	99.76	99.53	100.00	99.53
Iva2	**99.18**	98.73	98.80	98.73	99.10	98.80
DNA polymearse	**99.28**	98.86	98.72	98.89	99.16	98.80
pTP	99.50	99.50	99.10	**99.55**	99.35	99.35
52 K	99.36	**99.84**	98.56	99.68	99.36	99.68
pIIIa	**99.55**	99.55	99.09	99.32	99.37	99.49
penton	**99.59**	98.44	97.74	99.36	99.59	97.62
pVII	99.50	98.66	99.00	**99.83**	**99.83**	99.16
V	**99.82**	98.38	98.01	98.83	99.46	98.83
pX	**100.00**	100.00	100.00	100.00	99.59	100.00
pVI	**99.60**	97.60	97.08	96.68	96.40	99.07
hexon	**99.79**	88.63	85.91	84.92	88.50	88.23
protease	99.19	98.05	97.40	**99.51**	97.56	98.05
DBP	**99.69**	97.11	96.67	96.98	96.86	97.61
100 K	**99.63**	98.15	97.40	98.35	97.65	98.85
pVIII	**99.71**	98.25	95.77	98.10	97.37	98.10
E3	**99.36**	82.95	84.41	82.97	82.97	82.88
fiber	**99.06**	74.28	76.39	85.44	85.31	74.39
E4	99.08	99.20	**99.50**	98.93	98.66	98.24
Complete genome	**99.39**	98.24	98.43	96.99	97.37	98.26

Note: The highest similarity value in each row is in boldface

### Recombination analysis

**Fig. 2:**
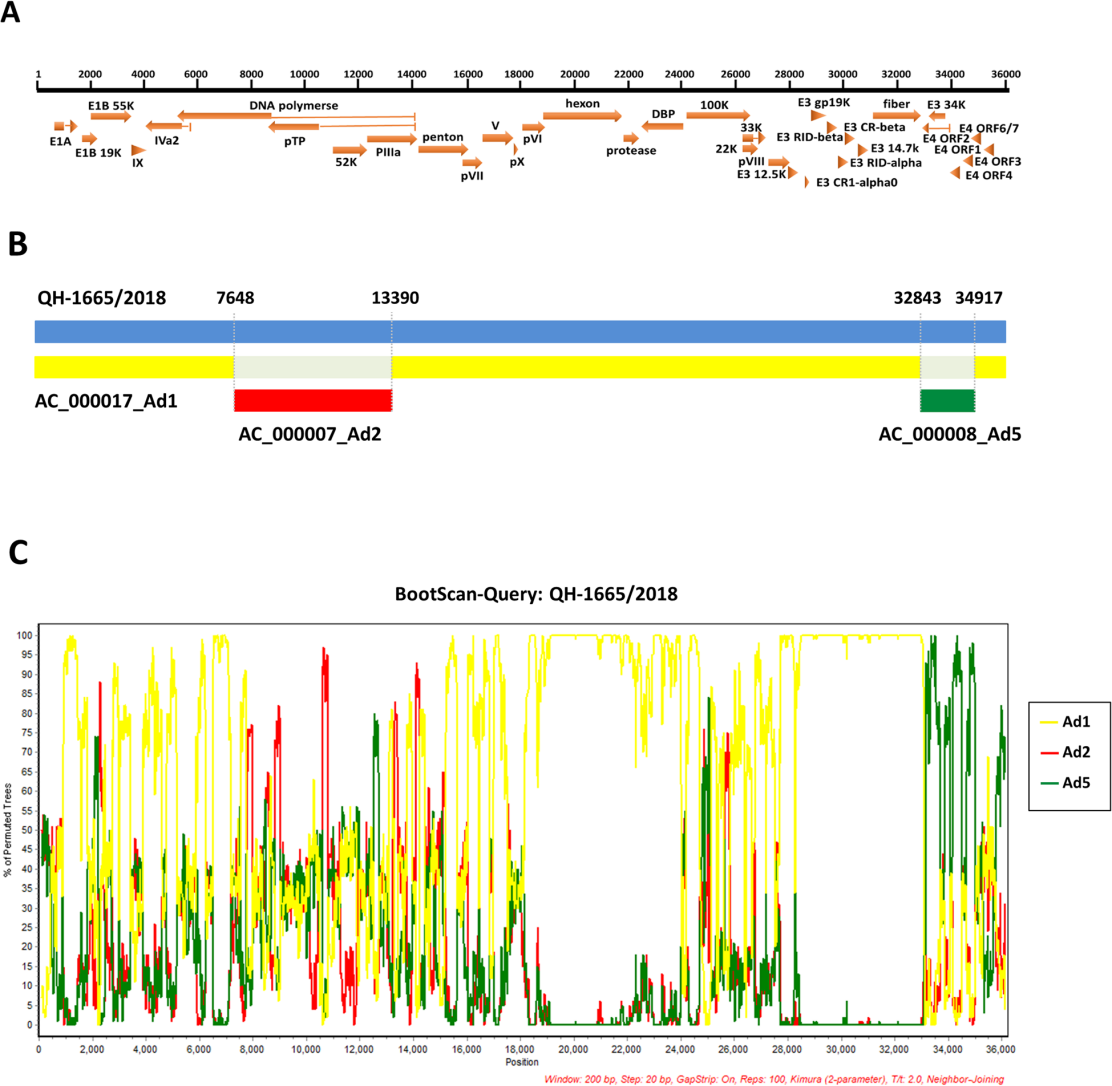
Genomic recombinant analyses of the complete genome of QH-1665/2018. **a** Genetic organization of QH-1665/2018. **b** Recombination events predicted in strain QH-1665/2018. QH-1665/2018 genome is shown with blue. The likely backbone is shown with yellow. Recombination event predicted by RDP4 are shown as red and green, respectively. Likely breakpoint positions are shown above the genome. **c** BootScan analysis of QH-1665/2018. QH-1665/2018 was used as the query sequence and compared with 3 representative strains of HAdV-C, including HAdV-1 (AC_000017.1), HAdV-2 (AC_000007.1) and HAdV-5 (AC_000008.1). The default setting of SimPlot software was used as follows: window size 200 bp, step size 20 bp. 100 replicates used, gap stripping, distance model (Kimura) and tree model (neighbor-joining)

To identify the recombination events within the genome of QH-1665/2018, recombination analysis was performed using the RDP4 package with multiple algorithms. Seven algorithms (RDP, GENECONV, BootScan, MaxChi, Chimaera, SiScan, 3Seq) were utilized to predict potential recombination events between the input sequences. The results indicated that it was a highly probable homologous recombinant resulting from HAdV-1 (AC_000017.1), HAdV-2 (AC_000007.1) and HAdV-5 (AC_000008.1) (Table 2). One recombinant event appeared with a beginning breakpoint at around 7648 (without gaps) and an ending breakpoint at around 13,390 (without gaps), with the major parent strain of HAdV-1 and a minor parent strain of HAdV-2, encompassing the genes pTP and 52 k as well as pIIIa partially. Another recombinant event appeared with a beginning breakpoint at around 32,843 (without gaps) and an ending breakpoint at around 34,917 (without gaps), with the major parent strain of HAdV-1 and a minor parent strain of HAdV-5, including most of E4 gene (Fig. 2a, b). BootScan analysis was then performed to confirm the recombination events within the genome of QH-1665/2018 by using SimPlot software (Fig. 2c).

**Table 2:** Algorithms of the RDP4 package used to predict the recombination event

Recombinant strain	Parent major/minor	Recombinant region in alignment	Model (average ***p***-value)						
			RDP	GENECONV	BootScan	MaxChi	Chimaera	SiScan	3Seq
QH-1665/2018	AC_000017_Ad1/AC_000007_Ad2	7655–13,405	2.45E-19	4.98E-06	1.44E-02	0.009607	1.37E-03	1.03E-02	4.08E-07
	AC_000017_Ad1/ AC_000008_Ad5	33,039–35,114	2.90E-19	3.79E-11	3.36E-08	5.00E-07	6.39E-05	2.09E-03	8.46E-10

**Fig. 3:**
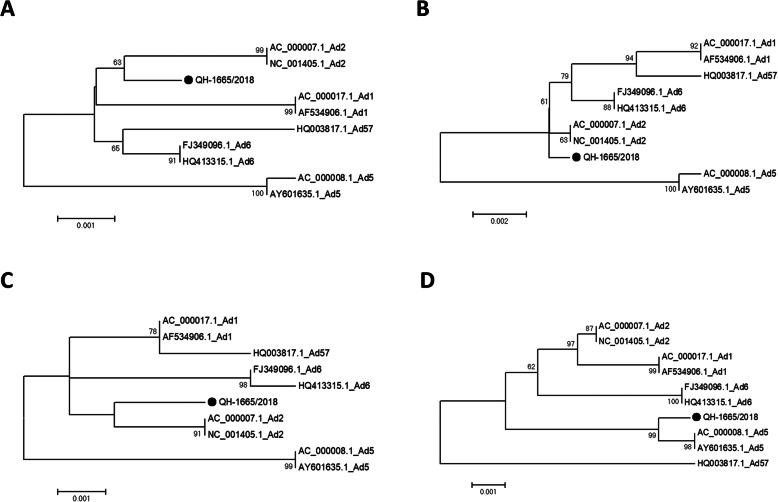
Phylogenetic analysis based on the recombinant region of QH-1665/2018. **a** pTP gene. **b** 52 K gene. **c** Partial pIIIa gene. D: E4 gene. The trees were constructed using the Maximum Likelihood (ML) method of MEGA 6.06 with 1000 bootstraps. QH-1665/2018 highlighted with a black dot was characterized in this study

The phylogenetic analysis also showed that recombinant region 1 (7648–13,390, pTP, 52 k and partial pIIIa) was clustered in HAdV-2, and recombinant region 2 (32843–34,917, E4) was clustered in HAdV-5, which coincided with previous recombination analysis (Fig. 3). The results re-confirmed that QH-1665/2018 was a recombinant containing HAdV-1, HAdV-2 and HAdV-5 sequences.

## Discussion

Recombination is common and important for the evolution of adenoviruses, which can drive the production of new adenoviruses genotypes [11]. HAdV-B and HAdV-C are the epidemic strains, causing respiratory infections in China [9, 12, 13]. However, few observations on HAdV-C recombinants have been reported compared to HAdV-B [14, 15]. Recently, new adenovirus genotypes have been increasingly recognized based on whole genome sequencing [15, 16]. The previous studies showed that three recombinant HAdV-C strains (BJ04, BJ09 and CBJ113) have been identified, but with different recombination events [7, 17], which indicated that HAdV-C recombination was very complex.

In this study, through phylogenetic analysis of HAdV based on the complete genome and 3 major antigen genes (penton, hexon and fiber), QH-1665/2018 was clustered to HAdV-1 [P1H1F1], which could be considered the backbone of the prototype HAdV-1 genome. The comparative genome analysis between QH-1665/2018 and other HAdV-C strains displayed several genes that did not show the highest sequence similarity with HAdV-1, for example, 52 K showed the highest sequence similarity with HAdV-2 and E4 showed the highest sequence similarity with HAdV-5, which indicated QH-1665/2018 might be a recombinant. In addition, recombination analysis based on the RDP4 package and SimPlot software both showed that QH-1665/2018 had recombinant events involving HAdV-1, HAdV-2 and HAdV-5, which was re-confirmed by phylogenetic analysis. The recombination areas were located between 7648 and 13,390, which included pTP, 52 k and partially pIIIa, and between 32,843 and 34,917, which included most of E4. Interestingly, the recombination event occurred at pTP, 52 k, pIIIa and E4 of HAdV-1, but not in known recombination hotspots, such as the penton base, hexon, and fiber [18]. pTP and E4 are early genes, which are associated with viral DNA replication and transcription, and 52 k and pIIIa are late genes, which are associated with viral capsid formation [19, 20]. The function of the recombination events at these loci in HAdV-C evolution remains to be elucidated.

The recombination usually occurs between strains of the same species, and several adenovirus prototype strains were found to be intratypic recombinants [21]. Our previous study showed that HAdV-C (HAdV-1, HAdV-2, HAdV-5, HAdV-6) strains were circulating in Qinghai Province simultaneously [9], which could have provided the opportunity for intratypic recombinant events. It indicated that the recombinant HAdV-C types might have been circulating in Qinghai Province for a long time, but more sequences will be needed for further confirmation. It was reported that HAdV-1 and HAdV-2 could cause a higher morbidity rate than HAdV-5 and HAdV-6 did [7]. Whether recombination would influence the virulence, pathogenicity and clinical characteristics of HAdV strains should be investigated in the future.

## Conclusion

We showed that QH-1665/2018 was a recombinant HAdV-C strain, it arose through the recombination of three HAdV genotypes-HAdV-1, HAdV-2 and HAdV-5. Our results suggest that HAdV-C recombinant might be circulating in Qinghai Province, but large-scale molecular epidemiological investigation of HAdV-C recombination is needed to confirm this. Corresponding prevention and control strategies should be taken into consideration in future work.

## Data Availability

The data supporting the conclusions of this article are included within the article.

## Abbreviations

CPE: Cytopathic effect
HAdVs: Human adenovirus
HEp-2: Human laryngeal epidermoid cancer cells
ML: Maximum Likelihood
RDP: Recombination Detection Program
